# Inflammation Targeting‐Triggered Healing Hydrogel for In Situ Reconstruction of Colonic Mucosa

**DOI:** 10.1002/advs.202411010

**Published:** 2025-01-15

**Authors:** Gaoxian Chen, Xinyi Li, Wei Xu, Haoze Wang, Yichao Jiang, Ruofan Shi, Chengying Zhu, Zeyu Xiao

**Affiliations:** ^1^ Collaborative Innovation Center for Clinical and Translational Science Department of Pharmacology and Chemical Biology, & Institute of Molecular Medicine School of Medicine Shanghai Jiao Tong University Shanghai 200025 P. R. China; ^2^ School of Life Sciences Shanghai University Shanghai 200444 China

**Keywords:** healing hydrogels, inflammation adhesive, inflammatory bowel disease, mucosal injury

## Abstract

Inflammatory bowel disease (IBD) is characterized by intestinal mucosal damage that exacerbates inflammation and promotes disease recurrence. Although hydrogel‐based therapies have shown potential for mucosal repair, challenges remain due to inadequate targeting and low hydrogel density, leading to ongoing infiltration of harmful substances and delayed mucosal healing. In this study, an inflammation‐targeting‐triggered healing hydrogel (ITTH hydrogel) is developed, composed of polyvinyl alcohol‐alginate microgels (PALMs) and a cyclodextrin polymer crosslinker (CPC). This hydrogel specifically targets inflamed colonic sites and crosslinks in situ to form a dense network. The results demonstrate that the ITTH hydrogel adheres effectively to inflamed colonic tissue in both IBD mouse models and human samples. The dense crosslinked network acts like the mucosal barrier, preventing the penetration of detrimental substances such as bacteria and small molecules, thereby protecting the underlying mucosal tissue. Furthermore, the ITTH hydrogel significantly improved therapeutic outcomes in mice with dextran sulfate sodium (DSS)‐induced colitis. These findings suggest that the ITTH hydrogel is a promising candidate for in situ reconstruction of colonic mucosa and the treatment of IBD.

## Graphical abstract

Inflammatory bowel disease (IBD) involves mucosal damage that exacerbates inflammation and recurrence. This study introduces an inflammation‐targeting‐triggered healing (ITTH) hydrogel, which specifically targets inflamed colonic tissue and undergoes in situ re‐crosslinking to form a dense barrier. This barrier prevents harmful substance infiltration, protects mucosal tissue, and significantly improves therapeutic outcomes.

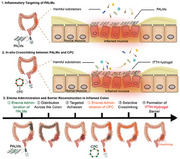

## 1 Introduction

Inflammatory bowel disease (IBD) mainly includes ulcer disease (UC) and Crohn's disease (CD).^[^ ^1^ ^]^ It is a chronic, immune‐mediated, inflammatory disease of the digestive tract.^[^ ^2^ ^]^ Over the past three decades, the incidence of IBD has been rising globally, posing a significant public health challenge.^[^ ^3^ ^]^ Mucosal injury is an important event in the pathogenesis of IBD,^[^ ^4^ ^]^ resulting in mucosal dysfunction, heightened intestinal permeability, and increased disease activity, significantly impacting the quality of life of patient.^[^ ^5^ ^]^ Currently available treatments, including oral medications or anti‐inflammatory enemas, are associated with notable side effects such as severe diarrhea and systemic immune disorders, which greatly limit their efficacy and application.^[^ ^6^ ^]^ Consequently, there is an urgent need to develop novel therapeutic strategies that not only repair the mucosal barrier but also minimize adverse effects.^[^ ^7^ ^]^

In recent years, hydrogels, as new materials for alleviating IBD, have received widespread attention.^[^ ^8^ ^]^ Commonly used materials include polymers such as hyaluronic acid, alginate, chitosan, cyclodextrin, inulin, and agarose.^[^ ^9^ ^]^ These are valued not only for their excellent biocompatibility but also for their abundant functional groups, which facilitate various chemical modifications and structural adjustments. Various hydrogel formulations, including polysaccharide hydrogels, and protein hydrogels, have been developed to facilitate drug release, and establish a physical mucosal barrier, contributing to the alleviation of IBD.^[^ ^9^, ^10^ ^]^ However, the suboptimal targeting of hydrogels synthesized in vitro to inflammatory sites of the colon may result in reduced bioavailability, which limits its application in delivery.^[^ ^11^ ^]^ The development of inflammation‐targeting hydrogels based on surface interactions represents a notable improvement in targeting.^[^ ^12^ ^]^ However, the unregulated hydrogel gaps result in a inadequate hydrogel layer coverage, leading to the ingress of harmful substances through the gaps and hindering effective mucosal recovery. In addition, loaded drugs may also cause side effect.^[^ ^13^ ^]^

**Figure 1:**
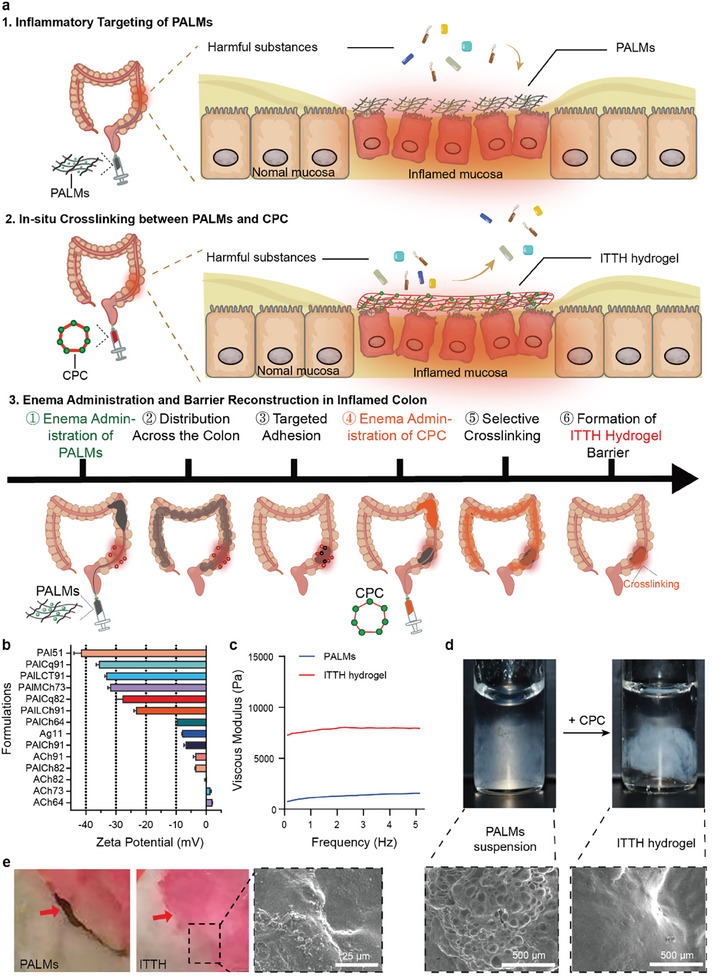
Characterization of ITTH hydrogel. a) Schematic Illustration of in situ reconstruction of colonic mucosa. The process includes three steps: First, PALMs target the inflamed colonic mucosa. Second, upon the addition of CPC, PALMs undergo crosslinking to form ITTH hydrogel, a large lamellar hydrogel that covers the entire inflamed mucosal surface, providing a protective barrier to the inflammation sites in the colon. Third, this illustration outlines the six‐step process of ITTH hydrogel formation for treating inflamed colonic tissue. PALMs are administered via enema, distribute across the colon, and selectively adhere to inflamed regions. A second enema introduces CPC, which crosslinks with PALMs at these sites, forming a ITTH hydrogel barrier that supports healing and resists degradation, ensuring targeted, long‐lasting retention for effective IBD treatment. b) Zeta potential of various formulations of polyvinyl alcohol‐based microgels. pAl51: Polyvinyl alcohol: Alginate = 5:1 (v/v, below); PAlCq91: (5:1):Cq = 9:1, Cq = chitosan quaternary (1.78%); PAlLCT91: (5:1):CTAB = 9:1; PAlMCh73: (5:1):MCh = 7:3, MCh = medium viscosity chitosan (1.0%); PAlCq82:(5:1):CQ = 8:2; PAlLCh91: (5:1):LCh = 9:1, LCh = low viscosity chitosan (1.5%); PAlCh64: (5:1):Ch = 6:4, Ch = chitosan hydrochloride (2%); Ag11. Agarose (0.8%); PAlCh91: (5:1): Ch = 9:1; ACh91: Ag:Ch = 9:1; PAlCh82: (5:1):Ch = 8:2; ACh82: Ag:Ch = 8:2; ACh73: Ag:Ch = 7:3; ACh64: Ag:Ch = 6:4. All results were obtained in pH 7.4 of artificial colonic fluid. *n* = 3 for each formulation; data are presented as mean ± SEM. c) Viscosity modulus of ITTH hydrogel and PALMs. d) Properties of ITTH hydrogel before and after dropwise addition of CPC and observations. The image on the bottom shows the successful adhesion of the lamellar PALMs gel formed after dropwise addition of CPC between two slides and the lamellar gel structure observed under eletron microscopy. e) Crosslinking interface formed by two PALMs after the addition of CPC, with Rhodamine B added to one of the PALMs for observation purposes under electron microscopy.

In this study, we designed an “inflammation‐targeting triggered healing hydrogel (ITTH hydrogel)”, which not only achieves high selectivity for colitis but also re‐crosslinks in situ, forming a densely interconnected hydrogel network layer that effectively prevents the invasion of harmful substances. (**Figure** **1**a). The ITTH hydrogel comprises two parts: the first part consists of alginate and PVA, both highly biocompatible. They were chosen for their excellent gel‐forming and re‐crosslinking properties, as well as their highly negative charge, which aids in targeting and adhering to sites of colonic inflammation; the second part is a cyclodextrin polymer crosslinker (CPC). Upon successful adhesion of polyvinyl alcohol‐alginate microgels (PALMs) to the inflamed tissue, the addition of CPC induces in situ crosslinking, forming the ITTH hydrogel with a robust crosslinked network. Compared to traditional methods, our ITTH hydrogel exhibits increased density as a barrier, thus effectively blocks the penetration of harmful bacteria and small molecules.

Our in vitro and in vivo studies show that the ITTH hydrogel exhibits excellent targeting and adhesion to inflamed colonic tissues in both IBD mouse models and UC patient biopsies. Moreover, it exhibits superior therapeutic effects compared to the commonly used mucosal repair agent bismuth potassium citrate (BPC) in dextran sulfate sodium (DSS)‐induced colitis mice, evidenced by reduced disease activity and enhanced mucosal healing. By forming a dense protective barrier through in situ crosslinking, it effectively shields the mucosa from harmful substances, acting like a synthetic mucosal layer. This innovative enema‐based hydrogel administration offers precise adhesion to inflamed sites and distinguishes itself from conventional therapies through targeted delivery and superior barrier function. The ITTH hydrogel holds significant potential for improving treatments of colonic diseases characterized by mucosal damage.

## 2 Results and Discussion

### 2.1 Characterization of ITTH Hydrogel

To achieve the high targeting of the hydrogel to the inflammatory site, we designed PALMs as the first part, and then to achieve in situ crosslinking in the inflammatory site, we added CPC as the second part. The two parts combine to form ITTH hydrogel. For the first part, inspired by the fact that negatively charged substances can specifically target the inflammatory sites of exposed positively charged substances,^[^ ^12^ ^]^ we designed a variety of different formulations of negatively charged microgels and measured their potentials to get the best targeting capacity of microgels (Figure 1 and Table S1, Supporting Information). The results of zeta potential showed that when the volume ratio of polyvinyl alcohol‐alginate mixture was 5:1(PAL51), the negative charge was always maintained in artificial colon fluid, which was ≈−41.6 mV, while other formulations were less negatively charged, with some even showing positive values, indicating that PAL51 has the best targeting ability. Polyvinyl alcohol is a biocompatible synthetic polymer known for its hydrophilic ‐OH group.^[^ ^14^ ^]^ Alginate, extracted from brown algae or bacterial cell walls, is also biocompatible and porous. Still, for effective use, it requires crosslinking, commonly with non‐toxic Ca^2+^.^[^ ^15^ ^]^ Then we choose polyvinyl alcohol‐alginate mixture as the next object of study. After CaCl_2_ crosslinking, we obtained polyvinyl alcohol‐alginate microgels (PALMs) as the first part of our ITTH hydrogel system.

To enable in situ re‐crosslinking at the inflammatory sites in the colon, we then incorporated CPC as the crosslinking agent. CPC is synthesized from cyclodextrin and borax. Cyclodextrin is an amphiphilic molecule characterized by a hydrophobic cavity and a hydrophilic surface, making it suitable for constructing stable hydrogel networks.^[^ ^16^ ^]^ The dynamic boronic acid ester bonds formed between polyvinyl alcohol and borax are widely used in self‐healing hydrogels.^[^ ^17^ ^]^ By combining these properties, CPC can efficiently facilitate the re‐crosslinking process, ensuring a robust and dense network structure within the ITTH hydrogel.

To confirm the successful re‐crosslinking of PALMs into ITTH hydrogel, Figure 1c showed that the initial viscosity modulus of ITTH hydrogel was ≈8 times higher than that of PALMs. This significant increase indicated the formation of a stable re‐crosslinked network, which is crucial for effectively covering damaged areas of the intestinal wall and underscores its potential protective role. The rapid crosslinking ability of PALMs was demonstrated in Figure S1 (Supporting Information) by observing morphological changes shortly after the addition of CPC. Within 5 s, microgels began to change, and by 13 s, they had fully aggregated and transitioned into a distinct white color, signifying complete re‐crosslinking. This swift transformation highlights the efficiency of the re‐crosslinking process, essential for timely therapeutic action. In addition, Movie S1 (Supporting Information) also intuitively observed that when CPC was added to the PALMs microgel, the ITTH hydrogel was formed immediately.

Electron microscopy provided further insights into the morphological changes during re‐crosslinking. As shown in Figure 1d, the transition from subspherical structures to flaky formations confirmed the quick crosslinking ability of PALMs to form ITTH hydrogel, which was significant for subsequent applications. Notably, an initial gap observed between two PALMs disappeared after crosslinking with CPC, as depicted in Figure 1e, confirming the formation of a continuous hydrogel network capable of adhering to and protecting inflamed intestinal sites.

Assessing the gelation process for different ingredient combinations, Figure S2 (Supporting Information) demonstrated that only the combination of PVA and alginate with CaCl₂ and CPC led to successful and complete ITTH formation. This finding emphasizes the importance of combining all these components for effective gelation and crosslinking. The ability to control microgel dimensions by adjusting sieve mesh sizes (50, 80, 100, 150, and 200 mesh), as shown in Figure S3 (Supporting Information), enables precise tuning of PALMs size and structure, allowing customization for specific therapeutic needs. This innovative approach, combining targeted adhesion and controlled crosslinking, highlights the significance of ITTH hydrogel in localized therapeutic delivery for intestinal diseases.

### 2.2 Inflammatory Targeting Characteristics and Safety Profile of ITTH Hydrogel in IBD Mice

**Figure 2:**
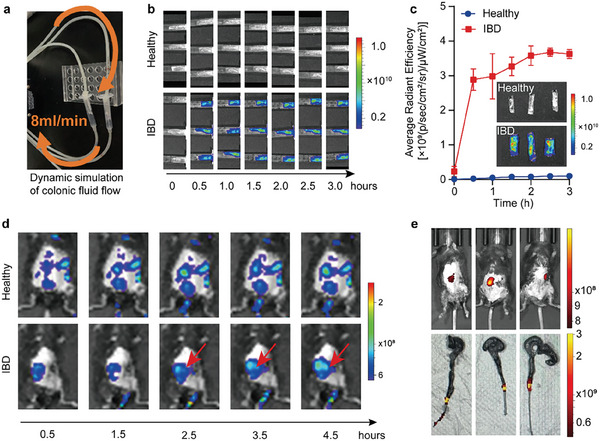
Inflammatory site targeting of ITTH hydrogel. a) Schematic illustration of the peristaltic pump loop. b) Fluorescence images of isolated colon tissues at different time points by using the peristaltic pump loop. c) Quantitative fluorescence intensity analysis on the surface of intestinal tissues over time for both groups in (b), with an inset displaying final IVIS fluorescence imaging; *n* = 3 for each formulation; data are presented as mean ± SEM. d) IVIS imaging results of healthy mice and IBD mice enriched with PALMs suspension at different times. The red arrows indicate localized aggregation of microgels in the intestine of IBD mice. e) in vivo IVIS imaging of fixed‐point modeled IBD mice with PALMs aggregation after 4.5 h (upper panel) and corresponding ex vivo IVIS imaging (lower panel), confirming targeted localization at inflamed sites.

We first analyzed the adhesive properties of PALMs ex vivo using custom‐built peristaltic pump loop (**Figure** **2**a). The Cy5‐labeled PALMs circulated in the syringe containing the isolated intestinal segments of healthy and IBD mice for 3 h. The fluorescence was observed every 0.5 h. Circulation flow rate of 8 mL min^−1^ simulated human intestinal fluid flow.^[^ ^18^ ^]^ As revealed in Figure 2b,c The fluorescence signal of the PALMs retained after circulation flow in IBD mice was ≈70 times higher than that of mice in the healthy group at 0.5 h. At the same time, the fluorescence signal became stable after 0.5 h, indicating that the PALMs would not detach from the intestinal wall with the flow of intestinal fluid. The above demonstrates that PALMs preferentially target the inflamed mucosa of colitis mice and has strong adhesion.

We explored the influence of PALMs' size and structure on their adhesion properties by extruding them through sieves of varying mesh sizes (50, 80, 100, 150, and 200 mesh). Fluorescence images demonstrated that PALMs of different sizes adhered to intestinal tissue with varying efficiencies over a 3‐h period (Figure S4a, Supporting Information). Smaller PALMs showed improved adhesion, with optimal results achieved at 100 mesh. Sizes below this threshold (100, 150, and 200 mesh) exhibited comparable adhesion without significant differences, suggesting a consistent performance at finer mesh sizes (Figure S4b, Supporting Information). This tunability is crucial for optimizing the therapeutic efficacy and ensuring adequate coverage of inflamed areas.

An essential consideration for any intestinal intervention is the risk of causing obstruction. To address this, we assessed the degradation behavior of ITTH hydrogel both in vitro and ex vivo. In simulated colonic fluid containing 20% mouse colon homogenates, Cy5‐labeled ITTH hydrogel showed gradual degradation over 48 h (Figure S5, Supporting Information), indicating that the hydrogel does not persist indefinitely within the intestinal tract. Ex vivo degradation studies using the peristaltic pump loop demonstrated a steady decrease in fluorescence intensity of the hydrogel under continuous flow conditions (Figure S6a–c, Supporting Information), confirming that the hydrogel degrades over time even in the dynamic environment of the intestine.

In vivo degradation studies further corroborated these findings. IVIS imaging of live animals demonstrated that the fluorescence signal of the Cy5‐labeled ITTH hydrogel diminished progressively over 48 h (Figure S7a–c, Supporting Information), confirming the hydrogel's biodegradability in a physiological environment. The controlled degradation profile ensures that the hydrogel remains at the site of inflammation long enough to exert its therapeutic effects up to 48 h, while minimizing the risk of intestinal obstruction due to hydrogel accumulation.

The ex vivo experiments further proved that PALMs preferentially adhered to the colonic mucosa of IBD mice. We examined the adhesion of PALMs to inflamed colon epithelium using the fixed‐point modeling procedure (Figure S8, Supporting Information). Mice were dissected, and injected 5%TNBS solution into their colon segment, then sutured. The inset in Figure S8 (Supporting Information) (right) displays the mucosal changes observed after removal and dissection of the modeled colon, showing darkened and damaged mucosa. This discoloration and injury indicate that the modeling was successful in inducing inflammation. Then the healthy mice and fixed‐point IBD model mice were given PALMs suspensions (labeled with Cy5) for 4.5 h after modeling. Observations revealed that, in fixed‐point IBD model mice, PALMs accumulated predominantly at the predetermined sites, which were aggravated with the passage of time due to continuous intestinal movement. Conversely, in healthy mice, the microgels were dispersed throughout the intestine (Figure 2d). It showed that the charge carried by the inflammatory site affected the distribution of PALMs, further illustrating the preferential targeting of inflammation by PALMs. When the intestinal tract was extracted from fixed‐point IBD model mice and IVIS imaging was performed, it was evident that the aggregation site of the microgel coincided with the previously determined inflammatory area (Figure 2e). In essence, these findings robustly confirmed that PALMs can proficiently target inflammatory areas of the intestinal tract of IBD mice. They remain anchored despite intestinal movements, progressively enhancing their coverage over inflamed areas. This lays the foundation for subsequent in situ re‐crosslinking and the study of ITTH hydrogel functions.

Collectively, these results confirm that PALMs can effectively target and adhere to inflamed areas in the intestinal tract of IBD mice. Their strong adhesion and ability to remain anchored despite intestinal movements facilitate progressive coverage of inflamed sites, setting the stage for subsequent in situ re‐crosslinking into ITTH hydrogel. The demonstrated biodegradability of ITTH hydrogel over 48 h addresses safety concerns by reducing the risk of intestinal obstruction, while providing sufficient residence time for therapeutic action. The capacity to control microgel dimensions and adhesion properties through mesh size adjustment, along with the favorable degradation profile, underscores the potential of ITTH hydrogel for safe and effective localized therapeutic delivery in intestinal diseases.

### 2.3 Inflammatory Targeting Characteristics of ITTH Hydrogel in Patients

**Figure 3:**
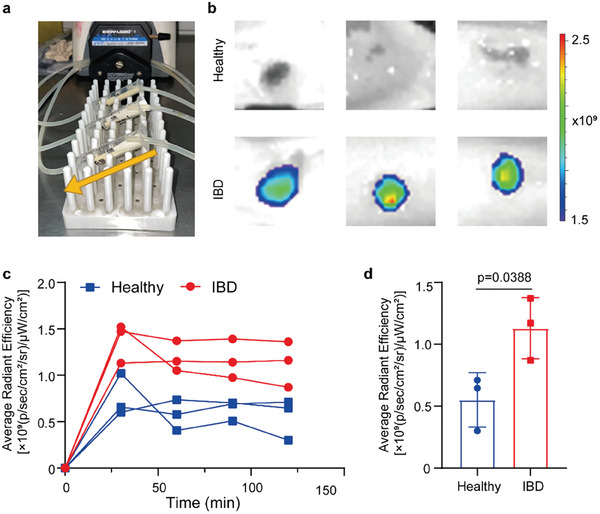
ITTH hydrogel specifically enriched in human inflammatory colon tissue. a) Photograph of Cy5 fluorescently labeled PALMs incubated with human colon tissue by a peristaltic pump device. b) IVIS images of colon tissues from different groups after 2 h incubating. c) Quantification evaluation of fluorescence signals of PALMs incubated with the colon tissues at different times. d) Mean fluorescence intensity values of Cy5‐labeled PALMs after 2 h of incubation with normal and inflammatory human colon tissues, shown with 95% confidence intervals and *p*‐values from *t*‐test analysis, *n* = 3.

To test the adhesion of PALMs to human colonic mucosa, we analyzed biopsy specimens from UC patients undergoing surveillance colonoscopy. We used the same peristaltic pump settings as shown in Figure 2a, and Cy5 pre‐labeled PALMs was circulated in syringes of healthy people and IBD patients for 2 h, respectively. The fluorescence was observed every 0.5 h. A circulation velocity of 8 mL min^−1^ simulated human intestinal fluid flow with three pathways independent of each other and only one piece of tissue incubated in each pathway at a single time (**Figure** **3**a). We observed a significant enrichment of PALMs in inflammatory colon tissue compared to normal colon tissue after 2 h incubation (Figure 3b). Specimens from inflamed sites retain significantly more fluorescence than specimens from normal sites; more than two‐fold difference between inflamed and normal biopsy specimens (Figure 3d). Finally, we plotted a line graph of the mean fluorescence intensity values on normal and inflammatory colon tissues after each 0.5 h incubation to visualize the change in the degree of enrichment of PALMs (Figure 3c). Taken together, consistent with our mouse data, PALMs preferentially adhere to biopsy specimens from human inflammatory colon tissue rather than normal colon tissue.

### 2.4 ITTH Hydrogel Protects Intestinal Mucosa

PALMs can adhere to the inflammatory site in the colon, but some harmful bacteria, proteins, and small molecules can still penetrate the intestinal mucosal barrier due to the low density after targeting, causing recurrence of inflammation. Therefore, we added CPC to form ITTH hydrogel between the dispersed PALMs to prevent the penetration of harmful substances.

**Figure 4:**
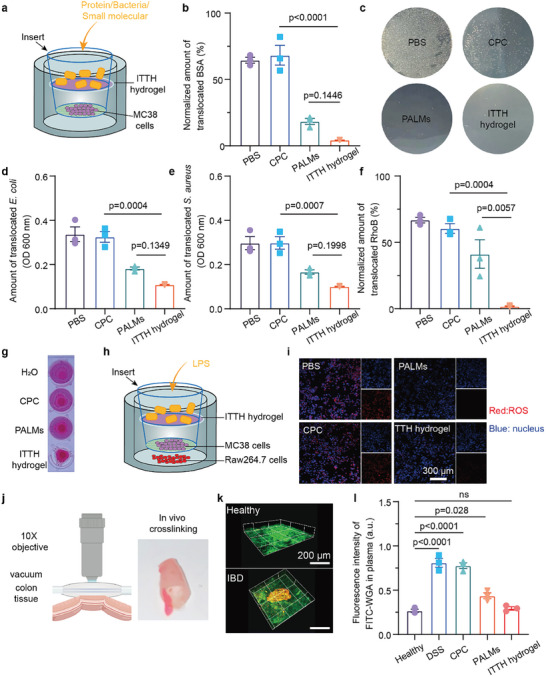
Protective effects of ITTH hydrogel on colonic mucosa. a) Schematic representation of in vitro ITTH hydrogel crosslinked and permeability experiments on protein, bacteria, or small molecular. b) Results of normalized values of BSA protein permeation for each group. c) The results of the experiment on the permeability of ITTH hydrogel to *E. coli* in vitro. d) Permeation quantification of *E coli* after ITTH hydrogel membrane formation in vitro. e) Permeation quantification of *S. aureus* after ITTH hydrogel membrane formation in vitro. f) Experimental results of normalized values of RhoB permeation for each group. g) Photographs of the results of in vitro ITTH hydrogel permeability experiments to the small molecule RhoB after film formation. h) Schematic representation of the permeability experiments on LPS antigen. i) Fluorescence imaging diagram of the permeability assay to LPS antigen after in vitro ITTH hydrogel formation. j) Schematic diagram of ITTH hydrogel aggregation in intestinal inflammatory sites of mice after crosslinking. k) 3D images of the ITTH hydrogel adhesion of in mice of different groups intestinal mucosa. Green: mucus stained with FITC‐wheat germ agglutinin. Red: PALMs stained with Cy5. l) FITC fluorescence intensity of blood samples. Data are shown as Mean ± SD (*n* = 3). Statistical significance in (b), (d), and (e) was determined using one‐way ANOVA with Fisher's least significant difference as a post hoc test.

Subsequently, we evaluated the impact of crosslinked networks on the permeability of intestinal harmful substances. The semipermeable membrane lined with MC38 cells simulates the intestinal wall as a platform for PALMs accumulation and crosslinking in vitro (**Figure** **4**a).^[^ ^19^ ^]^ Various substances like RhoB molecules, BSA protein, *E. coli*, *S. aureus*, and LPS were used to evaluate membrane permeability. With PALMs crosslinking by CPC, there was a marked decrease in standardized permeation values for BSA and RhoB molecules protein relative to their respective control groups (Figure 4b,f,g). Visualization showed the red RhoB solution retained atop the semi‐permeable membrane in the experimental group, while it permeated to the bottom chamber in control groups (Figure 4g). It shows that the crosslinked network of ITTH hydrogel successfully prevents the penetration of RhoB molecules and has a strong protective film effect.

For bacterial permeation assays involving *E. coli* and *S. aureus*, we quantified bacteria by measuring the turbidity of the bottom chamber solution post a 24‐h incubation. The pictures showed the colonies formed on the solid medium in each group of lower solution (Figure 4c; Figure S9, Supporting Information). Findings highlighted that ITTH hydrogel significantly curtailed bacterial permeation in comparison to controls (Figure 4d,e). Utilizing the ROS‐inducing properties of LPS, a bacterial lipopolysaccharide, we designed experiments to assess the permeability of ITTH hydrogel to LPS. As shown in Figure 4h,i, red fluorescence indicated DHE‐labeled reactive oxygen species charactering the degree of activation of Raw264.7 cells and fluorescence blue indicated DAPI‐labeled Raw264.7 cell nuclei. Fluorescence imaging confirmed that the endotoxin permeability decreased significantly with or without adding CPC. To sum up, when PALMs aggregate on semi‐permeable membranes and crosslink with CPC to form ITTH hydrogel, they significantly reduce the penetration of several substances, which means that they may mimic intestinal barrier function. Two‐photon fluorescence imaging confirmed the accumulation of PALMs in inflammatory intestinal mucosa and the formation of hydrogel blocks on localized inflammatory intestinal sections after the introduction of CPC into IBD mice. This is the crosslinked network formed by CPC crosslinking PALMs (Figure 2j,k).

Finally, to further explore the permeability of ITTH hydrogel, we assessed the fluorescence intensity of FITC in the blood. ITTH hydrogel was introduced into IBD mice for in vivo experiment and then add FITC‐dextran. Blood samples were subsequently analyzed for FITC fluorescence intensity. The increase of FITC blood fluorescence indicated increased intestinal penetration, which means a decrease in intestinal mucosal integrity. Results depicted 60 min post crosslinking, the FITC fluorescence intensity in ITTH hydrogel group was markedly lower than that in control group and PALMs group. Moreover, it was comparable to the FITC intensity in the blood of healthy mice post FITC‐dextran introduction (Figure 4l). This underscores that our ITTH hydrogel effectively mitigated the intestinal wall permeability of IBD mice, restoring it to levels seen in healthy mice.

### 2.5 Therapeutic Efficacy of ITTH Hydrogel in DSS‐Induced IBD Mice

**Figure 5:**
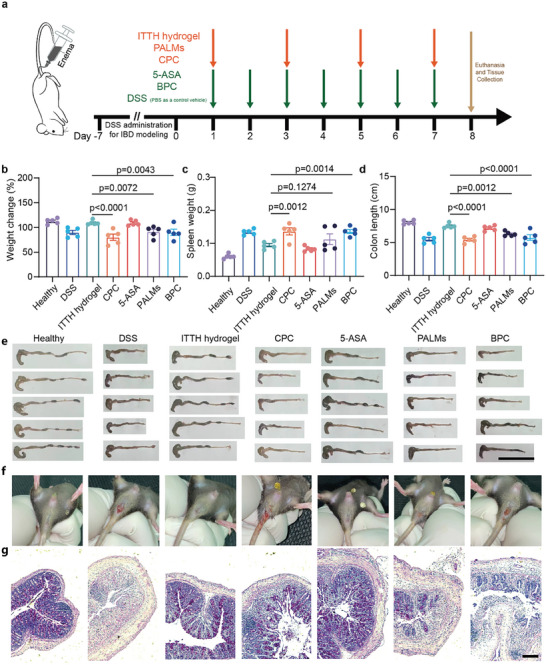
Assessment of treatment effects on a DSS‐induced mouse model of colitis. a) Timeline of the experimental design showing treatment schedules and key events. DSS was administered by free drinking from day −7 to day 0 to induce IBD in mice. Beginning on day 1, ITTH hydrogel treatment (comprising PALMs and CPC) was administered every other day, while the positive controls (5‐ASA and BPC) and vehicle control (PBS) were given daily throughout the treatment period. b) Change in body weight at the completion of treatment, showing percentage weight change relative to baseline. c) Spleen weights as an indicator of splenomegaly, used to assess systemic inflammation. d) Quantification of colon length, an indicator of colonic inflammation and tissue damage. e) Representative photograph of colon length for each group; scale bar = 5 cm. f) Anal appearance at the end of treatment, illustrating visual signs of inflammation or recovery. g) Representative PAS‐stained histological sections of colon tissue, with purple‐stained regions representing PAS‐positive areas indicating mucosal integrity; scale bar = 100 µm. Statistical analysis was performed using one‐way ANOVA with Fisher's least significant difference as a post hoc test. Data are presented as mean ± SEM, with a sample size of *n* = 5 per group.

To evaluate the therapeutic potential of ITTH hydrogel in inflammatory bowel disease (IBD), we utilized a dextran sulfate sodium (DSS)‐induced colitis mouse model. The experimental design and treatment schedules are outlined in **Figure** **5**a. DSS was administered daily from day −7 to day 0 to induce colitis in the mice. Beginning on day 1, ITTH hydrogel treatment—comprising PALMs and CPC—was administered every other day. Positive controls, including 5‐Aminosalicylic acid (5‐ASA), the first‐line clinical drug for IBD, and Bismuth potassium citrate (BPC), which forms microgels with charge properties comparable to PALMs under alkaline conditions, were administered daily throughout the treatment period. PALMs or CPC alone were used as negative controls to assess the specific benefits of the ITTH hydrogel, and phosphate‐buffered saline (PBS) served as the vehicle control.

Treatment with ITTH hydrogel markedly alleviated disease activity indicators in the colitis mice. Mice treated with ITTH hydrogel‐maintained body weight more effectively than those in the PALMs, CPC, and BPC groups (Figure 5b), indicating a significant mitigation of systemic disease symptoms. Spleen enlargement, a marker of systemic inflammation, was substantially reduced in the ITTH hydrogel group, comparable to the reduction observed with 5‐ASA treatment (Figure 5c). Compared to other treatment groups, the ITTH hydrogel group showed more significant alleviation of colon length shortening caused by colonic fibrosis (Figure 5d,e), suggesting superior efficacy in reducing colonic inflammation and promoting tissue healing.

Anal examination of the mice further supported these findings. Mice in the DSS, CPC, and BPC groups exhibited significant anal prolapse and bleeding, whereas those treated with ITTH hydrogel displayed normal anal morphology similar to healthy controls (Figure 5f). This observation underscores not only the therapeutic effectiveness but also the safety profile of ITTH hydrogel. Histopathological analyses reinforced the therapeutic benefits of ITTH hydrogel. Periodic acid‐Schiff (PAS) staining revealed a significant increase in mucin‐secreting goblet cells in the ITTH hydrogel group compared to other treatments (Figure 5g), indicating enhanced mucosal barrier restoration.

Crucially, we achieved direct in vivo visualization of ITTH hydrogel formation at inflamed colonic sites, providing compelling evidence of its in situ gelation and mucosal reconstruction capabilities. Previously, assessments relied on fluorescence imaging to infer gel formation. In this study, the formation of a visibly white gel was apparent to the naked eye (Figure S10 and Movie S2, Supporting Information), marking the first direct observation of in situ gelation in vivo. The black dotted areas in Figure S10 (Supporting Information) highlight the extent of ITTH hydrogel coverage over the affected tissue, demonstrating robust adhesion and mechanical strength. This in situ formed gel remained intact during colonic peristalsis without causing intestinal obstruction, effectively reconstructing the colonic mucosa.

Furthermore, colonoscopy, the clinical gold standard for diagnosing IBD,^[^ ^20^ ^]^ demonstrated that mice treated with ITTH hydrogel had regular colonic folds with intact mucosal coverage, in contrast to the severe mucosal defects observed in the DSS and CPC groups and the mild inflammation in the PALMs and BPC groups (Figure S11, Supporting Information). This observation reinforces the histopathological findings and underscores the efficacy of ITTH hydrogel in restoring colonic mucosal integrity.

In summary, ITTH hydrogel exhibits significant therapeutic efficacy in the DSS‐induced colitis model, surpassing the effectiveness of the first‐line clinical drug 5‐ASA and other control treatments. The direct observation of its in situ gel formation at inflamed sites not only validates its mechanism of action but also highlights its potential as an innovative approach for mucosal barrier reconstruction in IBD therapy.

## 3 Conclusion

In conclusion, we successfully developed a novel method for hydrogel delivery to ameliorate colon inflammation through in situ re‐crosslinking to reconstruct the mucosal barrier. Through the first part's PALMs targeting capability and adhesion toward inflammation sites and the second part's CPC in situ gelation feature, ITTH hydrogel demonstrated excellent properties. Our selected PALMs showed effective inflammation targeting and adhesion both in vitro and in vivo, and this was further validated in UC patient samples. Due to the inclusion of CPC in the second part, ITTH hydrogel re‐crosslinked in situ at inflammation sites. The re‐crosslinking ITTH exhibited resistance to the invasion of bacteria, small molecules, and proteins, thereby alleviating prolonged stress on the intestinal mucosa and facilitating the repair and regeneration of damaged mucosal tissue. In in vivo treatment studies, ITTH hydrogel demonstrated promising therapeutic outcomes, achieving comparable efficacy to the first‐line clinical drug 5‐ASA and showing superior effects compared to PALMs alone. This engineering strategy directly targets inflammation and reduces side effects associated with traditional drug treatments, making ITTH hydrogel a groundbreaking approach in long‐term management of IBD.

What truly sets our ITTH hydrogel apart is its unique, fully autonomous two‐tiered targeting and barrier‐reconstructing mechanism, which is an achievement no previous studies have reported.^[^ ^9^, ^21^ ^]^ Traditional targeting hydrogels have primarily functioned as drug carriers,^[^ ^9^, ^22^ ^]^ releasing medication upon electrostatic or ligand‐mediated interactions with inflamed sites.^[^ ^23^ ^]^ In contrast, our approach leverages electrostatic attraction to create an initial gel layer that adheres automatically to inflamed tissue, eliminating the need for complex drug loading and targeting ligands. In this system, PALMs act as “bricks,” autonomously locating and accumulating at inflammation sites, while CPC serves as the “cement,” crosslinking these PALMs in situ to form a stable, cohesive “patch” that effectively restores the mucosal barrier. This innovative design achieves a densely packed and resilient barrier, a capability previously unattainable in IBD treatment. Our in vivo studies confirmed this barrier‐reconstructing ability, with the ITTH hydrogel‐forming an integrated, crosslinked patch directly at inflammation sites. Additionally, our approach, grounded in the reliable principle of electrostatic attraction, is efficient, cost‐effective, and scalable, presenting a strong case for clinical translation. This breakthrough represents a novel, transformative strategy in colonic hydrogel therapy, offering significant advancements in the treatment of IBD.

## Conflict of Interest

The authors declare no conflict of interest.

## Supporting information



Supporting Information

Supplemental Movie 1

Supplemental Movie 2

## Data Availability

The data that support the findings of this study are available from the corresponding author upon reasonable request.
