# Supplementary material for: Inflammation Targeting‐Triggered Healing Hydrogel for In Situ Reconstruction of Colonic Mucosa
Source: Adv Sci (Weinh). 2025 Jan 15;12(10):2411010. doi: 10.1002/advs.202411010 (PMC11904975; doi:10.1002/advs.202411010)
Supplement: Supplementary file 1 — Supporting Information [file ADVS-12-2411010-s001.docx]

Supporting Information

**Inflammation Targeting-Triggered Healing Hydrogel for In-situ Reconstruction of Colonic Mucosa**

Gaoxian Chen, Xinyi Li, Wei Xu, Haoze Wang, Yichao Jiang, Ruofan Shi, Chengying Zhu, and Zeyu Xiao *

G. Chen, X. Li and W. Xu contributed equally to this work.

G. Chen, X. Li, H. Wang, Y. Jiang, R. Shi, C. Zhu, Z. Xiao

Collaborative Innovation Center for Clinical and Translational Science, Department of Pharmacology and Chemical Biology, & Institute of Molecular Medicine, School of Medicine, Shanghai Jiao Tong University, Shanghai 200025, P. R. China

W. Xu

School of Life Sciences, Shanghai University, Shanghai, 200444, China

E-mail: [zxiao@sjtu.edu.cn](mailto:zxiao@sjtu.edu.cn) (Z. Xiao)

**Experimental Section**

**Preparation of ITTH hydrogel****:** ITTH hydrogel was made from PALMs plus CPC. PALMs was prepared by mixing 10% 145k polyvinyl alcohol (PVA, Maclean's reagent) with 5% alginate (Alginate, Maclean's reagent) gel (5:1 v/v), immersed in saturated CaCl_2_ solution for 30min and filtered through a 100-mesh cell sieve. Then it was prepared by centrifugal washing with 2 x 10000rpm 1min. The prepared PALMs were dispersed in artificial colonic fluid. CPC was prepared from water-soluble cyclodextrin polymer and anhydrous borax in a ratio of 5:4. After centrifugation at 10,000 rpm, take it out and use it.

**Characterization of ITTH hydrogel:** To prepare negatively charged microgels, several different formulations based on Alginate and polyvinyl alcohol were designed and their zeta potential was measured to select the most negatively charged formulation as the microgels carrier for subsequent experiments. For the preparation of the microgels, the polymers were dissolved and mixed well, immersed in saturated CaCl_2_ solution for 30 min, filtered through a 100 mesh cell sieve, and washed twice by centrifugation (1 min, 10,000 rpm). The prepared microgels were dispersed in ACF and the corresponding zeta potentials were determined under these conditions. The final formulations used in experiments are presented in Table S1.

**Gelation process of different ingredients:** To examine the gelation process of various formulations, three groups were prepared: PVA only, Alginate only, and a combined PVA + Alginate formulation. In the PVA group, a 10% PVA solution (145k) was prepared and added to Tube 1. After 30 minutes, CaCl₂ was added to Tube 2 to observe potential gel formation; however, gel formation was unsuccessful in the PVA-only group, so further steps (Tubes 3 and 4) were not carried out. In the Alginate group, a 5% Alginate solution was added to Tube 1, and CaCl₂ was subsequently added to Tube 2, which induced initial gelation. The solution was then extruded through a 100-mesh cell sieve to form microgels (Tube 3). Finally, CPC was added to Tube 4, completing the crosslinking process. In the PVA + Alginate group, a mixture of 10% PVA and 5% Alginate (5:1 v/v) was placed in Tube 1, followed by CaCl₂ in Tube 2, which produced gelation. The solution was filtered through a 100-mesh sieve to form microgels in Tube 3, and CPC was then added to Tube 4, resulting in crosslinked microgels. Gel formation in each tube was visually assessed and compared across groups.

**PALMs morphology at different sieve mesh sizes:** To assess the morphology of PALMs (polyvinyl alcohol-alginate microgels) extruded through sieves of different mesh sizes, PALMs were prepared by mixing a 10% PVA solution with a 5% Alginate solution at a 5:1 v/v ratio, followed by immersion in a saturated CaCl₂ solution for 30 minutes. The mixture was then extruded through cell sieves of varying mesh sizes (50, 80, 100, 150, and 200 mesh) to obtain microgels with different diameters. Samples from each mesh size were examined and photographed under a microscope, and morphological differences in size and structure were documented.

**Adhesion of Cy5-labeled PALMs to intestinal tissue:** The adhesion of Cy5-labeled PALMs to intestinal tissue was analyzed *ex vivo*. PALMs were prepared as described and labeled with Cy5 dye, then extruded through cell sieves of 50, 80, 100, 150, and 200 mesh sizes. Freshly isolated sections of intestinal tissue were incubated with each group of PALMs in artificial colonic fluid at 37°C for 3 hours. Fluorescence images were taken at 0 and 3 hours to visualize PALM adhesion.

***In vitro* degradation of ITTH hydrogel in simulated colonic fluid**

To study the *in vitro* degradation of ITTH hydrogel, Cy5-labeled hydrogels were incubated in simulated colonic fluid. Cy5-labeled ITTH hydrogel samples were prepared and immersed in simulated colonic fluid containing 20% mouse colon homogenates. Samples were incubated at 37°C, and images were captured at 0, 6, 12, 24, 36, and 48 hours. Degradation was assessed by visual inspection of hydrogel structure over time, and degradation rates were determined by analyzing fluorescence intensity changes.

***Ex vivo* degradation analysis of ITTH hydrogel using peristaltic pump loop:** An *ex vivo* degradation assay was performed to assess the stability of ITTH hydrogel in a simulated colonic environment under flow conditions. Cy5-labeled ITTH hydrogel was prepared and placed in a custom-designed peristaltic pump loop filled with simulated colonic fluid containing 20% mouse colon homogenates. The peristaltic pump was set to operate at a flow rate of 8 mL/min to simulate colonic fluid dynamics. Fluorescence images of the hydrogel were captured at 0, 6, 12, 24, 36, and 48 hours. Images were analyzed to determine changes in fluorescence intensity, which served as an indicator of degradation. Quantification of average radiant efficiency was conducted to monitor the fluorescence over time, and relative radiant efficiency was calculated by normalizing against a blank background.

***In vivo* degradation of ITTH hydrogel:** *In vivo* degradation of ITTH hydrogel was assessed in live mice using IVIS imaging. Mice received an enema of Cy5-labeled ITTH hydrogel, which was administered directly into the colon. IVIS images were captured at 6, 10, 24, 36, and 48 hours post-administration to monitor hydrogel degradation *in vivo*. Images were processed to visualize degradation over time, and average radiant efficiency was quantified to determine the fluorescence signal intensity associated with the hydrogel. Relative radiant efficiency was normalized against a background signal.

***Ex vivo* adhesion assay:** We used a home-made intestinal fluid system to simulate human intestinal pH and intestinal fluid flow rate: a whole section of a healthy mouse colon was cut, the lining was unfolded and spread out in a tube along the longitudinal axis. Then the tube was connected to a peristaltic pump, 2 mL of Cy5-labeled PALMs were injected and the peristaltic pump was run at 8 ml/min for 2 h. The intestinal section was then subjected to IVIS Fluorescence imaging to observe and analyze the enrichment of the microgels. Experimental methods for UC patients’ biopsy samples are consistent with this.

***In vivo* adhesion assay:** C57BL/6J mice were randomly assigned into two experimental groups: (1) Healthy control group (no colitis); (2) Experimental colitis model group, which received 100 μL PALMs infusion per mouse. After treatment, mice were euthanized, and colonic tissues were collected for IVIS fluorescence imaging. Two intestinal segments from each mouse were fixed in 4% paraformaldehyde, frozen, and subsequently analyzed by fluorescence microscopy to evaluate microgel adhesion.

**Permeability assay of RhoB *in vitro*:** MC38 cells were laid on the lower layer of the semi-permeable membrane to simulate intestinal epithelium. 1.2 ml of PALMs artificial colonic suspensions were added on the semi-permeable membrane, left to settle naturally for 1 h and then 100 μl of 50% cyclodextrin aqueous solution was added and left for 30min to form a crosslinked membrane, as the experimental group, to test the permeability of the layer. Control group 1 added only 1.3 ml of water to the semi-permeable membrane; control group 2 added only 100 μl of 50% cyclodextrin aqueous solution to the semi-permeable membrane; control group 3 added only 1.2 ml of PALMs artificial colon fluid suspension to the semi-permeable membrane. To the upper layer of each group, 100 μl RhoB solution was added and the colour of the upper and lower liquid layers was observed for 10 h of release. The ratio of the fluorescence intensity of the lower layer solution RhoB after 10 h release to the fluorescence intensity of the upper layer solution RhoB at the initial moment was used as a standardized value for RhoB permeation.

**Permeability assay of BSA *in vitro*:** The semi-permeable membrane was prepared in the RhoB assay. 100 μl of 1 mg/ml BSA protein solution was added to the upper layer of each group, and the protein concentration in the solution was determined by the BCA protein quantification method. The ratio of the protein concentration in the lower layer of the solution after 10 h of release to the protein concentration in the upper layer of the solution at the initial moment was used as a standardised value for BSA protein permeation.

**Permeability assay of *E. coli* and *S. aureus* *in vitro*:** Using the same setup as described in permeability assays, *E. coli* or *S. aureus* bacterial suspensions were added to the upper layer of each group, and initial absorbance at 600 nm was standardized across groups. Bacterial permeation into the lower solution was quantified by measuring absorbance at 600 nm, indicating the bacterial load transferred across the semi-permeable membrane.

**Permeability assay of LPS antigen *in vitro*:** The lower layer of the semi-permeable membrane was lined with MC38 cells mimicking the intestinal epithelium and the bottom layer of the EP tube was lined with Raw264.7 cells, if the microgel was unable to resist the penetration of the LPS antigen into the lower layer, the LPS antigen would react with Raw264.7 cells to produce ROS. The nuclei of Raw264.7 cells at the bottom of the EP tubes were stained for DAPI and the ROS produced were stained for DHE. The extent to which Raw264.7 was activated was characterised by fluorescence microscopy, reflecting the amount of LPS antigen infiltration.

***In vivo* ITTH hydrogel permeability assay:** Mice were tested for colonic permeability after microgel enucleation: healthy mice were selected without microgel enucleation operation and 100 μL FITC-dextran was applied as a control; mice with experimental colitis were selected for microgel enucleation and 100μL 20% cyclodextrin solution was applied 2 h later and 100 μL FITC-dextran was applied 2 h later. Blood was immediately collected from the fundus venous plexus at 30 min, 45 min and 60 min after enema. 100 μl of blood was collected from each mouse and centrifuged in a centrifuge tube containing EDTA at 3500 rpm for 10 min. After centrifugation, 50 μl of supernatant was gently removed and aspirated for fluorescence signal intensity detection in an enzyme marker.

**Establishment of colitis model in mice:** C57 mice (weighing 19-20 g) aged 7 weeks were fed with 3% DSS (MP Biomedicals) for 7 days to induce DSS modeled mice. To induced acute colonic injury in mice by the method reported in the literature, mice were anaesthetized by inhalation of isoflurane, dissected, a segment of the proximal colon (10 mm internal diameter) was clamped with a circular forceps, a trinitrobenzene sulfonic acid (TNBS) solution (5%, 100 μL, Sigma) was injected into the lumen of the colon using a 29-gauge needle, held in the clamp for 2 minutes, and then released before the colon returned to the abdominal cavity. The incision was sutured. All mice were euthanized at the end of the experiment.

**Therapeutic efficacy of ITTH hydrogel:**

Induction of colitis and treatment administration:

Colitis was induced in C57BL/6J mice (7 weeks old, weighing 19–20 g) by administering 3% dextran sulfate sodium (DSS; MP Biomedicals) in drinking water from Day -7 to Day 0. Beginning on Day 1, mice were divided into seven groups: (1) Healthy (no DSS treated), (2) DSS (3% DSS only), (3) ITTH hydrogel (3% DSS + 455 mg/kg PALMs + CPC in 100 μL enema), (4) CPC (3% DSS + CPC in 100 μL enema), (5) 5-ASA (3% DSS + 100 mg/kg 5-ASA in 0.5% HPMC enema), (6) PALMs (3% DSS + 455 mg/kg PALMs in 100 μL enema), (7) BPC (3% DSS + 220 mg/kg BPC in enema). All treatments were continued until Day 8, at which point mice were euthanized for further analysis.

Body weight measurement:

Body weight was recorded at baseline and at the end of the treatment period. Changes in body weight were calculated as a percentage relative to baseline and used as an indicator of general health and inflammation severity in response to DSS-induced colitis and subsequent treatments.

Spleen weight analysis:

Upon euthanasia, spleens were harvested and weighed to assess splenomegaly, an indicator of systemic inflammation. Increased spleen weight is associated with heightened inflammatory responses, providing insight into the systemic effects of each treatment. Spleen weights were recorded and analyzed across groups.

Colon length measurement:

Colons were dissected and measured for length as an indicator of colonic inflammation and tissue damage. Shortening of the colon is commonly associated with inflammation severity in DSS-induced colitis models. Colon length was quantified for each group to assess the efficacy of treatments in reducing inflammation and preserving tissue integrity.

Gross morphology and anal appearance:

Representative photographs of dissected colons were captured for each group at the end of the experiment. A scale bar of 5 cm was used to ensure consistent measurement. Additionally, images of the anal region were taken to visually assess external signs of inflammation or recovery. Changes in anal appearance served as an auxiliary indicator of inflammation severity and therapeutic effect.

Histological analysis with PAS staining:

Colon tissue sections were subjected to PAS (Periodic Acid-Schiff) staining to evaluate mucosal integrity. PAS staining highlights mucosal areas with intact glycoconjugates, which appear as purple-stained regions. Histological sections were analyzed for PAS-positive areas to determine the extent of mucosal healing.

**Endoscopic observations on mice:** Colonoscopy in mice was performed using a COLOVIEW high-resolution mouse endoscopic system (Karl Storz, Germany) under 2% isoflurane anesthesia. Pig endoscopy was conducted with an Olympus CV-290 endoscope (Olympus) under xylazine hydrochloride anesthesia, carried out by an experienced operator. A blinded gastroenterologist evaluated colonoscopic images for statistical significance.

**Statistical analysis:** Two-tailed t-tests were used to compare differences between two experimental groups, and for studies of UC patients unpaired Student t-tests were used. One-way ANOVA with Tukey's post hoc test was used in experiments with multiple groups. p-values < 0.05 were considered statistically significant. Statistical analyses and graphs were performed using Prism 7.0.

**Abbreviations**

| **Abbreviation** | **Full Term** |
| --- | --- |
| IBD | Inflammatory Bowel Disease |
| UC | Ulcerative Colitis |
| CD | Crohn's Disease |
| ITTH hydrogel | Inflammation Targeting-Triggered Healing Hydrogel |
| PALMs | Polyvinyl Alcohol-Alginate Microgels |
| CPC | Cyclodextrin Polymer Crosslinker |
| TNBS | Trinitrobenzene Sulfonic Acid |
| DSS | Dextran Sulfate Sodium |
| BPC | Bismuth Potassium Citrate |
| 5-ASA | 5-Aminosalicylic Acid |
| PAS | Periodic Acid-Schiff (stain) |
| ACF | Artificial Colonic Fluid |
| IVIS | In Vivo Imaging System |
| FITC | Fluorescein Isothiocyanate |
| DHE | Dihydroethidium |
| PVA | Polyvinyl Alcohol |
| SA | Sodium Alginate |
| CaCl₂ | Calcium Chloride |
| LPS | Lipopolysaccharide |
| HPMC | Hydroxypropyl Methylcellulose |
| SD | Standard Deviation |
| SEM | Standard Error of Mean |
| ANOVA | Analysis of Variance |

**Table S1** Formulations screened for microgel carriers

| Name | Formulation (%, w/v) |
| --- | --- |
| PAl51 | 8.3% PVA + 0.8% sodium alginate |
| PAlCq91 | 7.6% PVA + 0.8% sodium alginate + 0.2% chitosan quaternary ammonium salt |
| PAlLCT91 | 7.6% PVA + 0.8% sodium alginate |
| PAlMCh73 | 6.1% PVA + 0.6% sodium alginate + 0.3% medium viscosity chitosan |
| PAlCq82 | 6.3% PVA + 0.6% sodium alginate + 0.4% chitosan quaternary ammonium salt |
| PAlLCh91 | 8.2% PVA + 0.5% sodium alginate + 0.1% low viscosity chitosan |
| PAlCh64 | 5.4% PVA + 0.5% sodium alginate + 0.7% chitosan hydrochloride |
| Ag11 | 0.8% agarose |
| PAlCh91 | 8.2% PVA + 0.5% sodium alginate + 0.2% chitosan hydrochloride |
| ACh91 | 0.7% agarose + 0.2% chitosan hydrochloride |
| PAlCh82 | 7.3% PVA + 0.5% sodium alginate + 0.4% chitosan hydrochloride |
| ACh82 | 0.6% agarose + 0.4% chitosan hydrochloride |
| ACh73 | 0.6% agarose + 0.6% chitosan hydrochloride |
| ACh64 | 0.5% agarose + 0.8% chitosan hydrochloride |


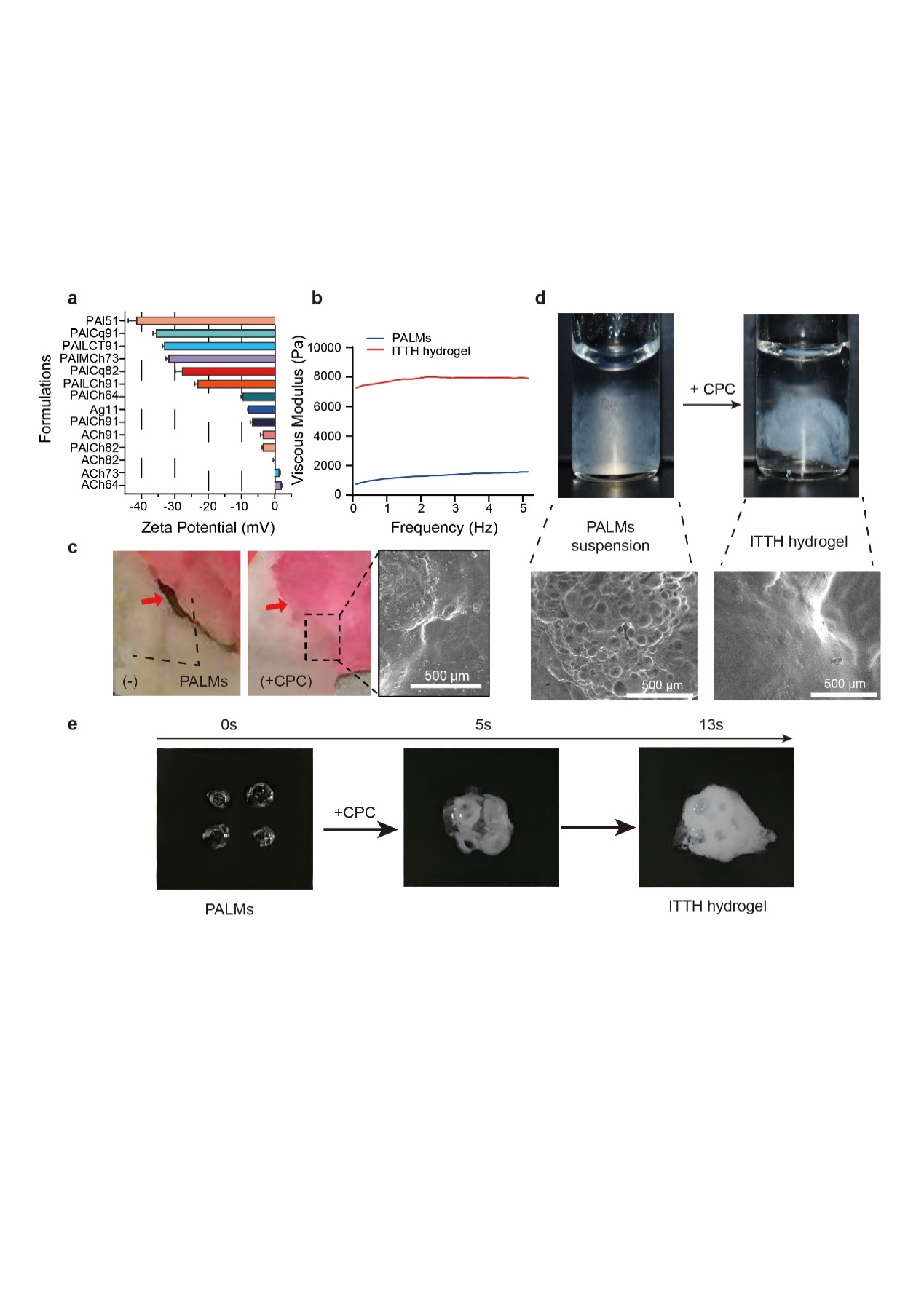


**Figure S1.** Formation process of ITTH hydrogel. Sequential images showing the transformation of PALMs into ITTH hydrogel upon CPC (crosslinking polymer component) addition. At 0 seconds, PALMs are prepared and isolated. At 5 seconds after adding CPC, initial crosslinking begins, creating a partially formed hydrogel. By 13 seconds, ITTH hydrogel forms as a cohesive structure, demonstrating the rapid crosslinking process.


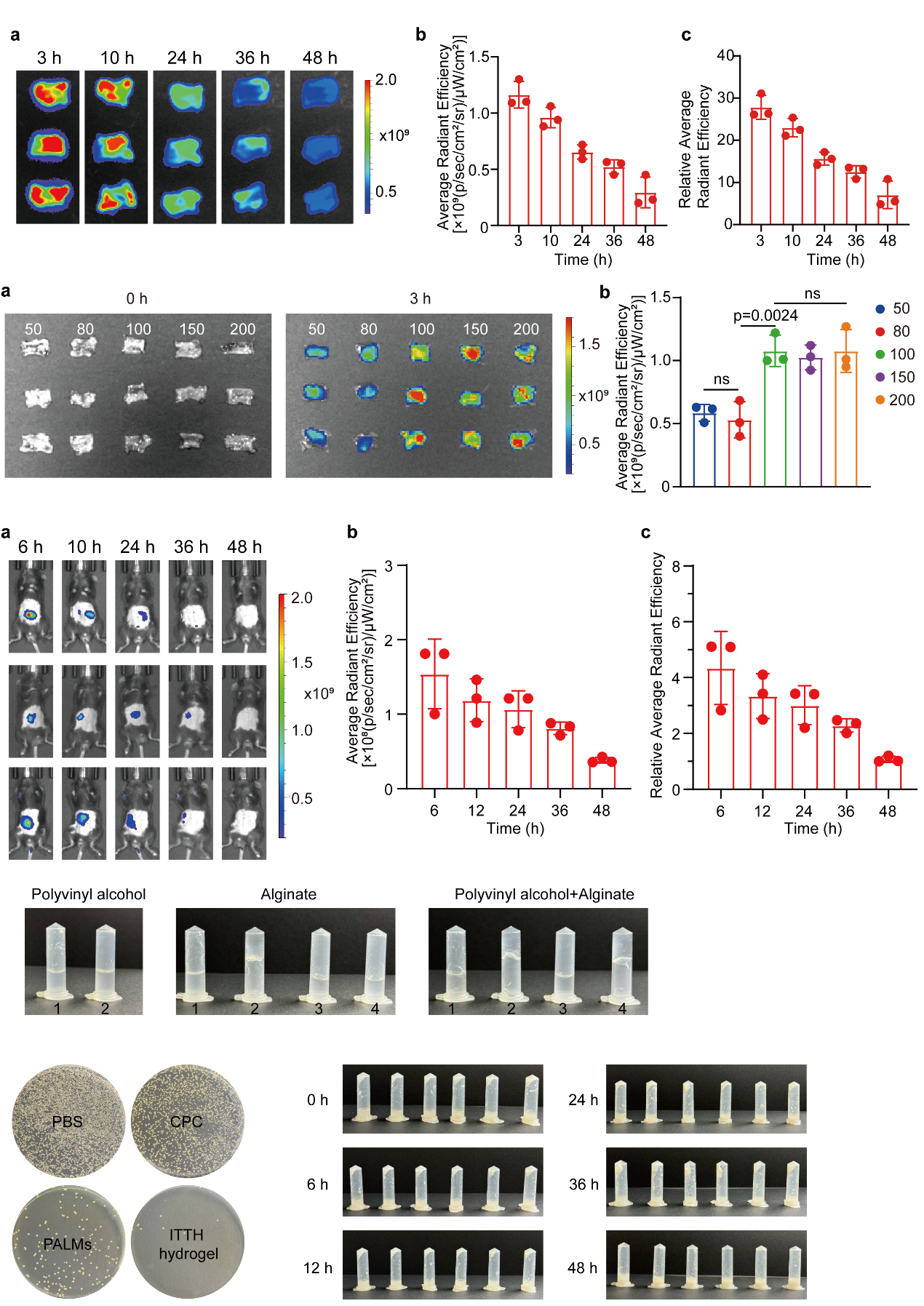


**Figure S2.** Gelation process of different ingredients. Comparison of gel formation across various formulations. Polyvinyl alcohol group: Only tubes 1 and 2 are present, as gel formation is unsuccessful. Alginate group: Sequential steps from Tube 1 to Tube 4 show gel formation. Polyvinyl alcohol + Alginate group: Shows full gelation process across all four tubes, indicating successful microgel formation with CaCl₂ and CPC additions. Tube 1 contains separate Polyvinyl alcohol and Alginate solutions, Tube 2 shows gel formation upon CaCl₂ addition, Tube 3 contains microgels formed by extrusion through a cell sieve, and Tube 4 demonstrates crosslinking with CPC.

**
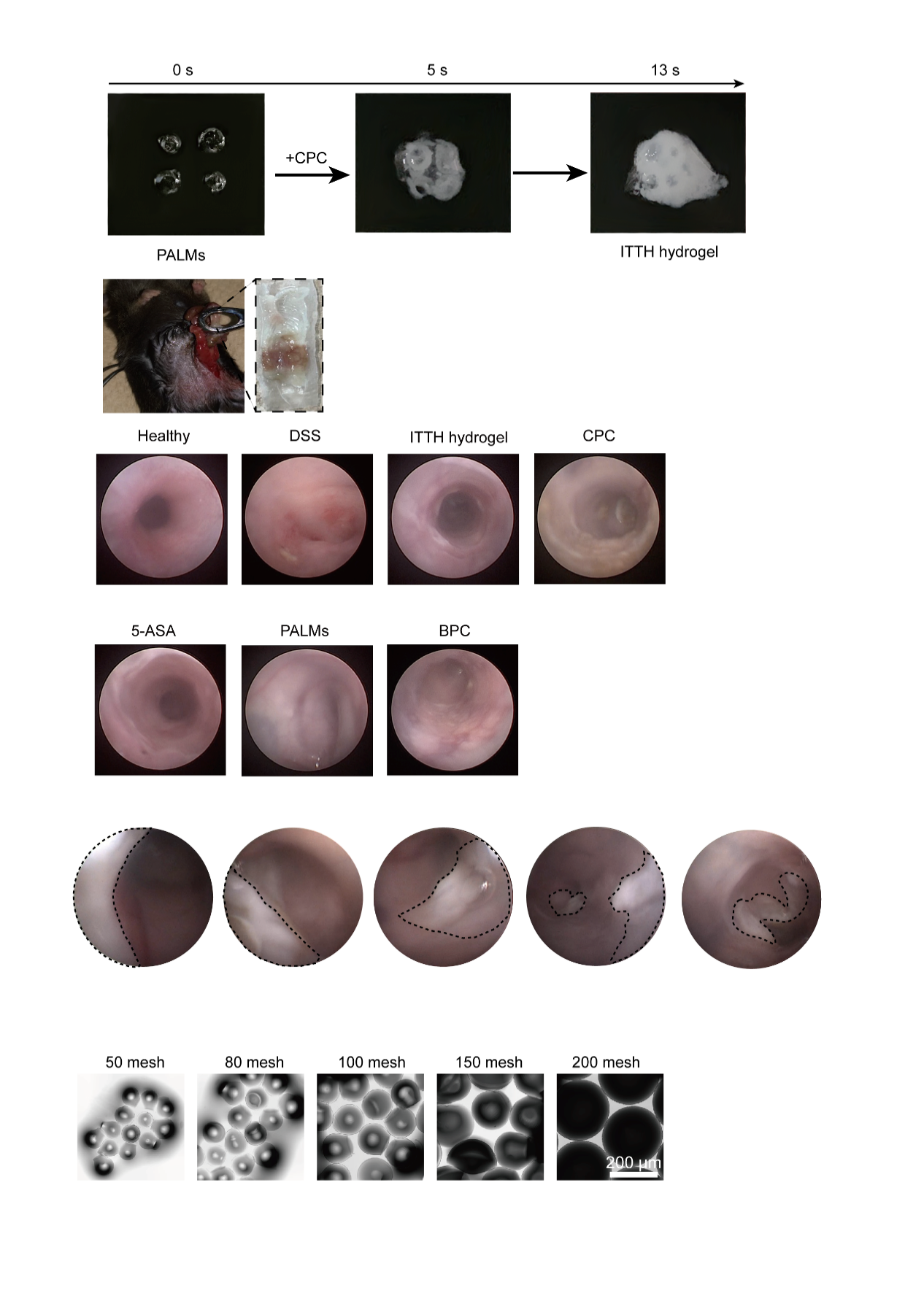
**

**Figure S3.** PALMs morphology at different sieve mesh sizes. Images of PALMs extruded through cell sieves with varying mesh sizes (50, 80, 100, 150, and 200 mesh). Scale bar = 200 μm.

**
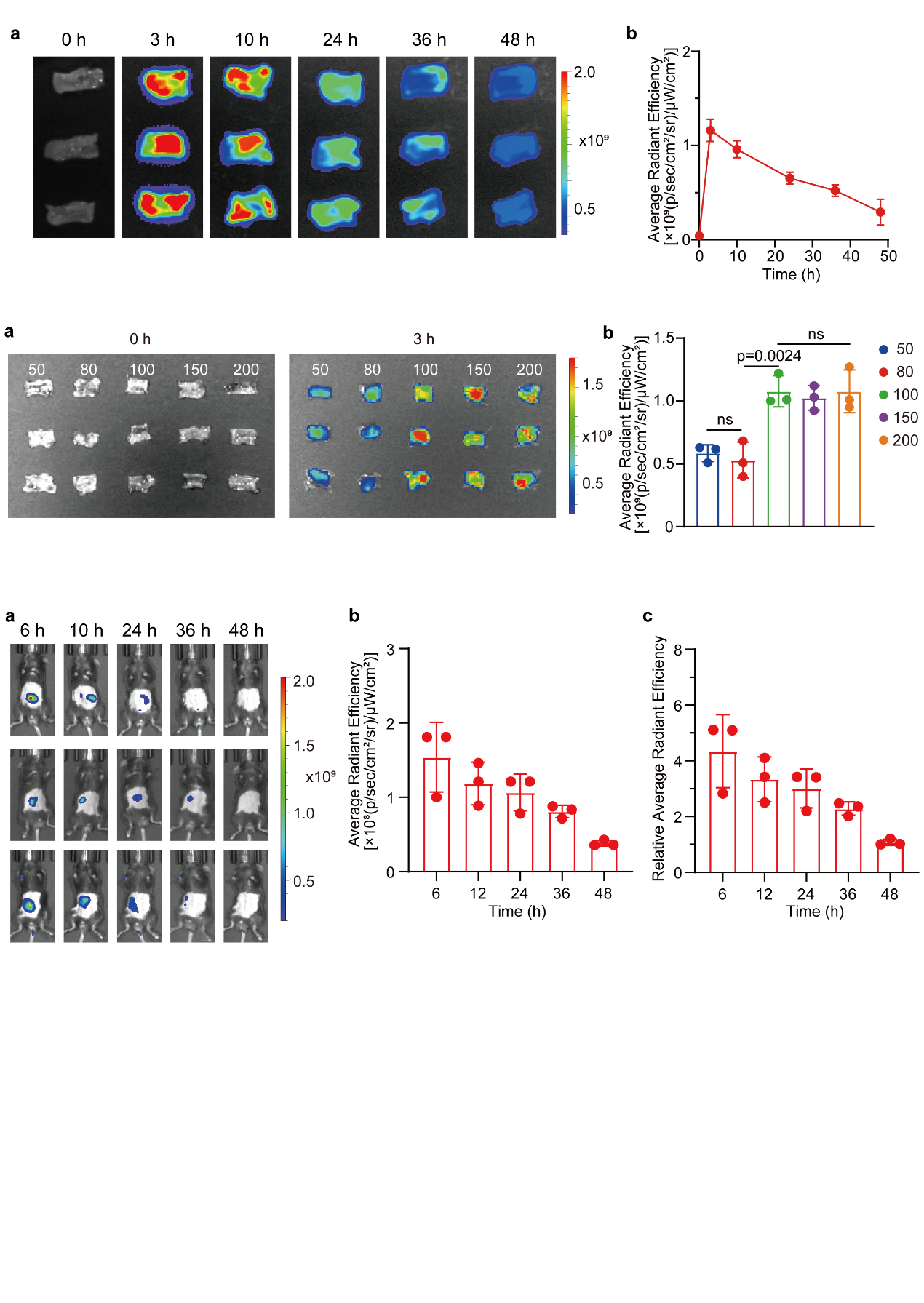
**

**Figure S4.** Adhesion of Cy5-labeled PALMs to intestinal tissue. (a) Fluorescence images showing adhesion of Cy5-labeled PALMs extruded through 50, 80, 100, 150, and 200 mesh sieves to intestinal tissue over 0 and 3 hours. (b) Quantitative analysis of fluorescence intensity for each group after 3 hours. Data are presented as mean ± SEM (n = 3), with statistical significance assessed via one-way ANOVA.

**
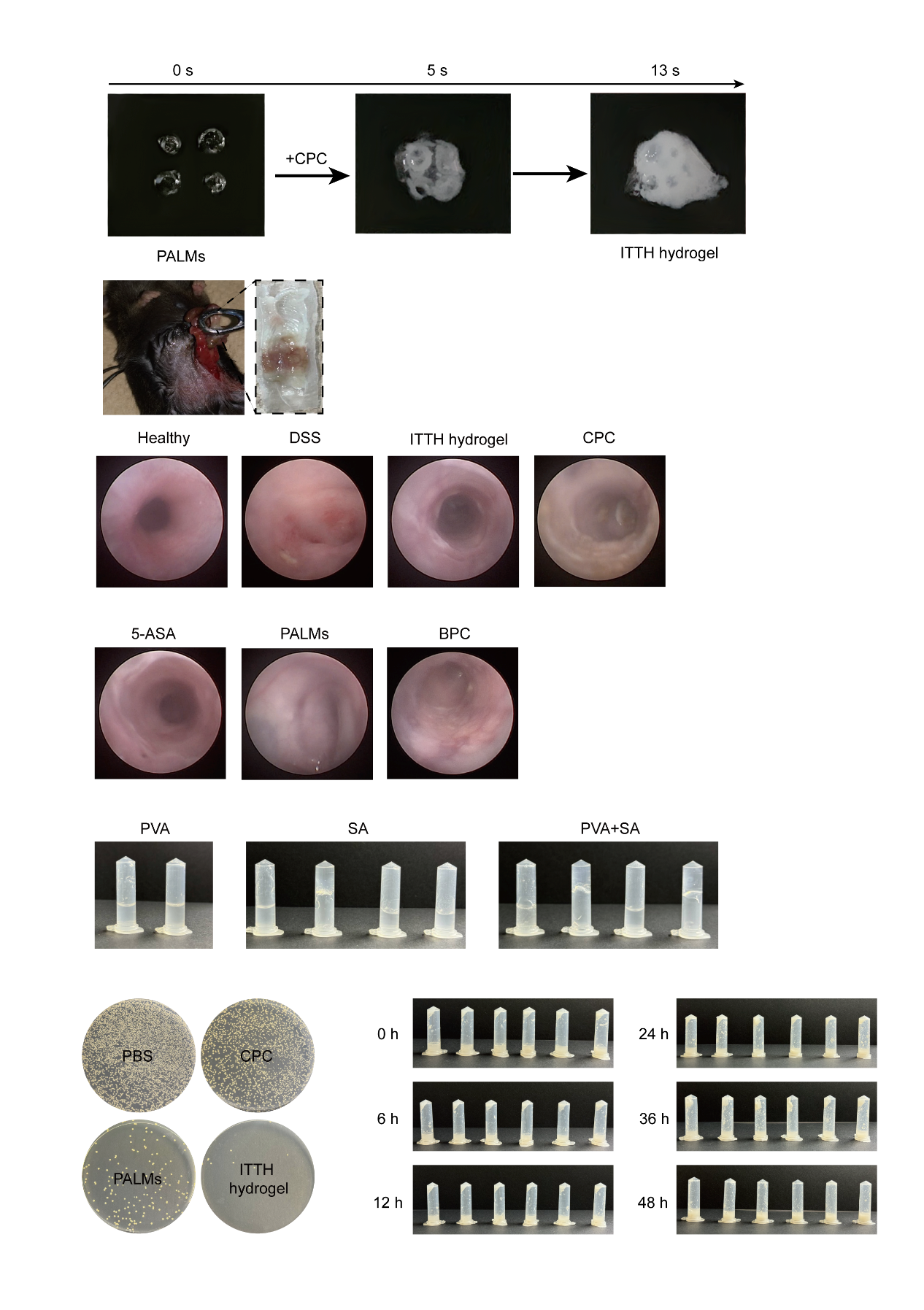
**

**Figure S5.** *In vitro* degradation of ITTH hydrogel in simulated colonic fluid. Cy5-labeled ITTH hydrogel is shown at 0, 6, 12, 24, 36, and 48 hours of degradation in simulated colonic fluid containing 20% mouse colon homogenates, indicating gradual breakdown of the hydrogel.


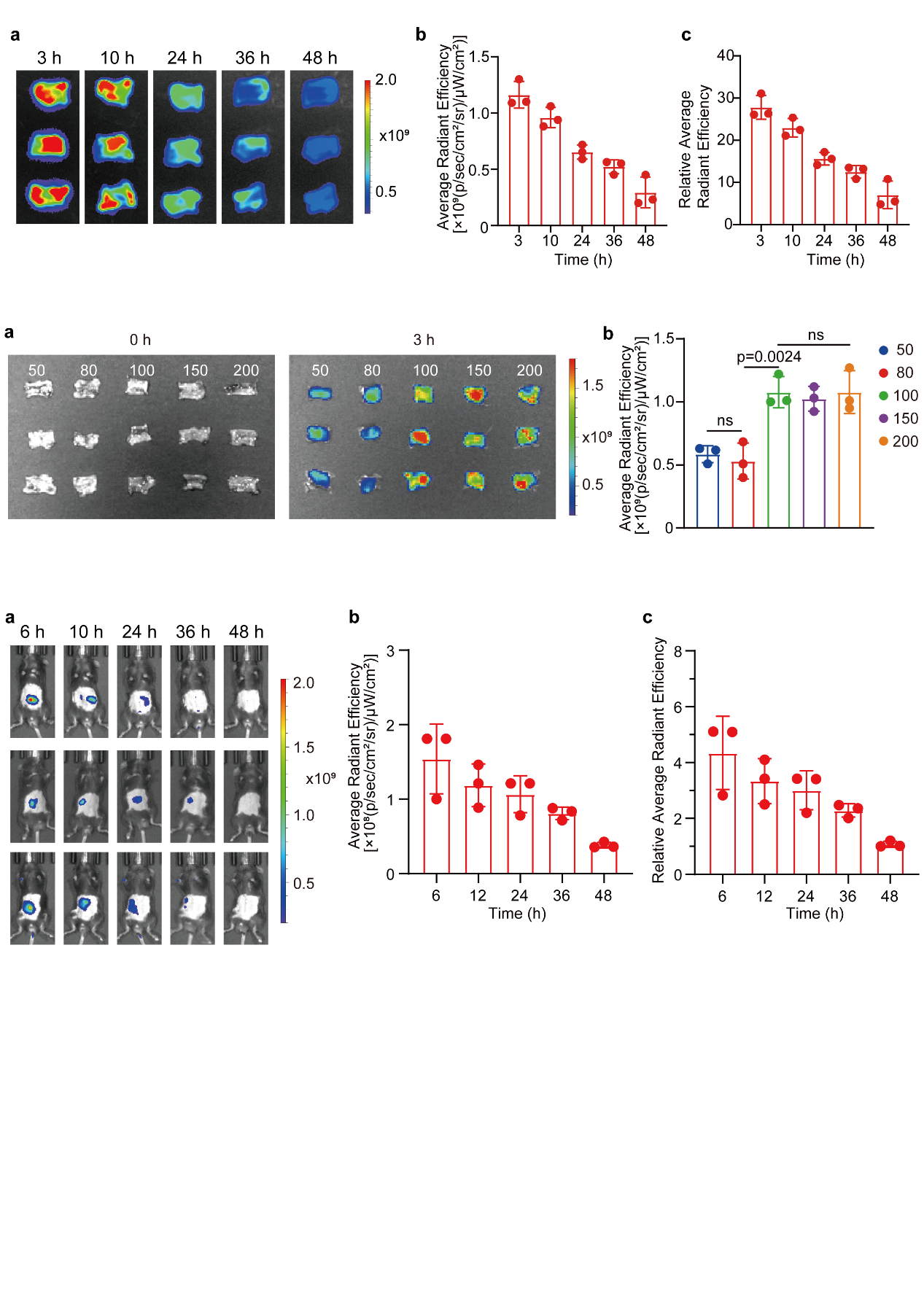


**Figure S6.** *Ex vivo* degradation analysis of ITTH hydrogel. (a) Fluorescence images showing the degradation of Cy5-labeled ITTH hydrogel in simulated colonic fluid under continuous flow using a peristaltic pump loop. (b) Quantification of average radiant efficiency over time. (c) Relative radiant efficiency to a blank background, reflecting the degradation rate. Data are shown as mean ± SEM.

**
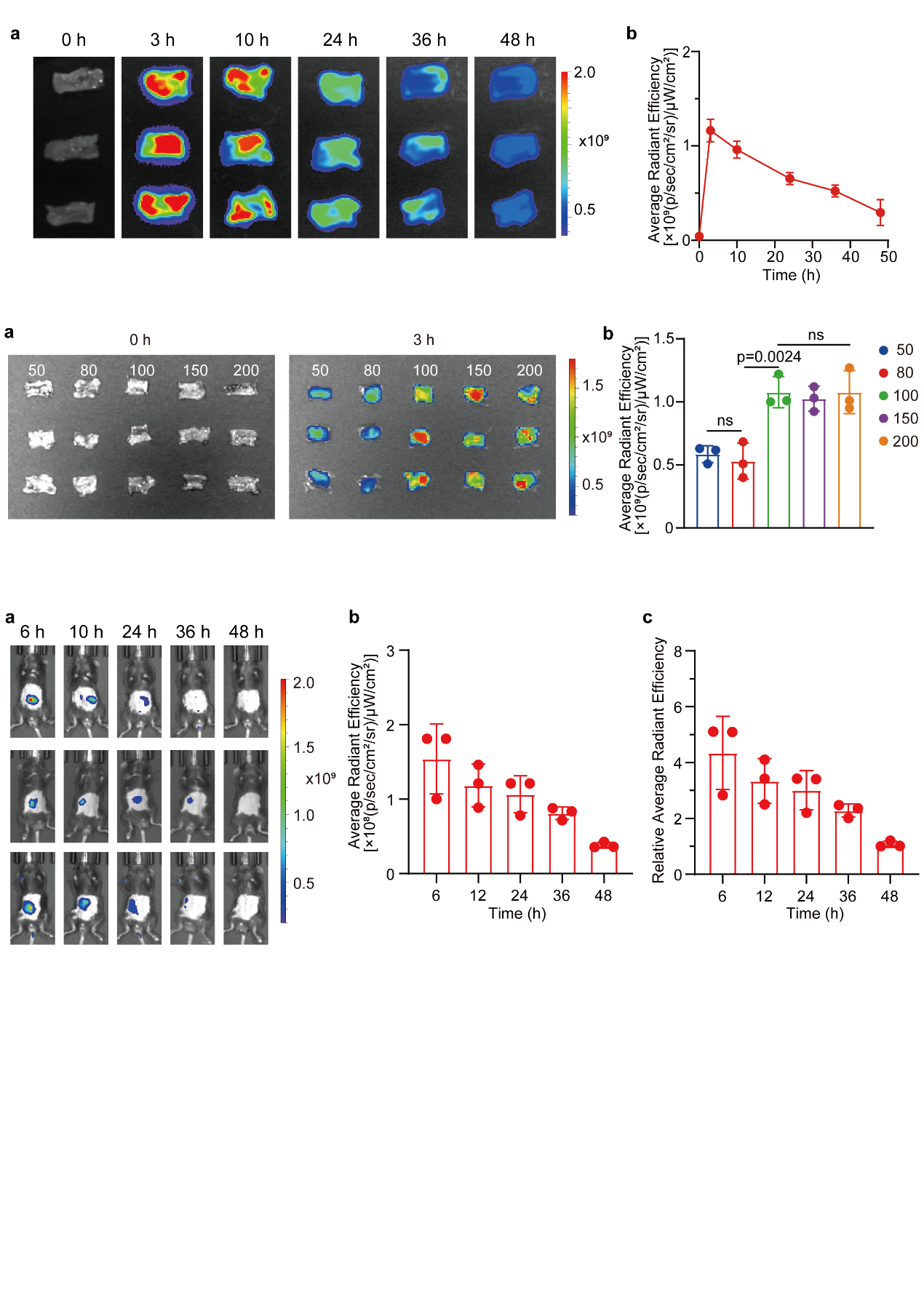
**

**Figure S7.** *In vivo* degradation of ITTH hydrogel. (a) IVIS images showing the degradation of Cy5-labeled ITTH hydrogel in live animals over 6, 10, 24, 36, and 48 hours. (b) Quantitative analysis of average radiant efficiency over time. (c) Relative radiant efficiency compared to background, demonstrating the hydrogel’s *in vivo* degradation profile. Data are presented as mean ± SEM.

**
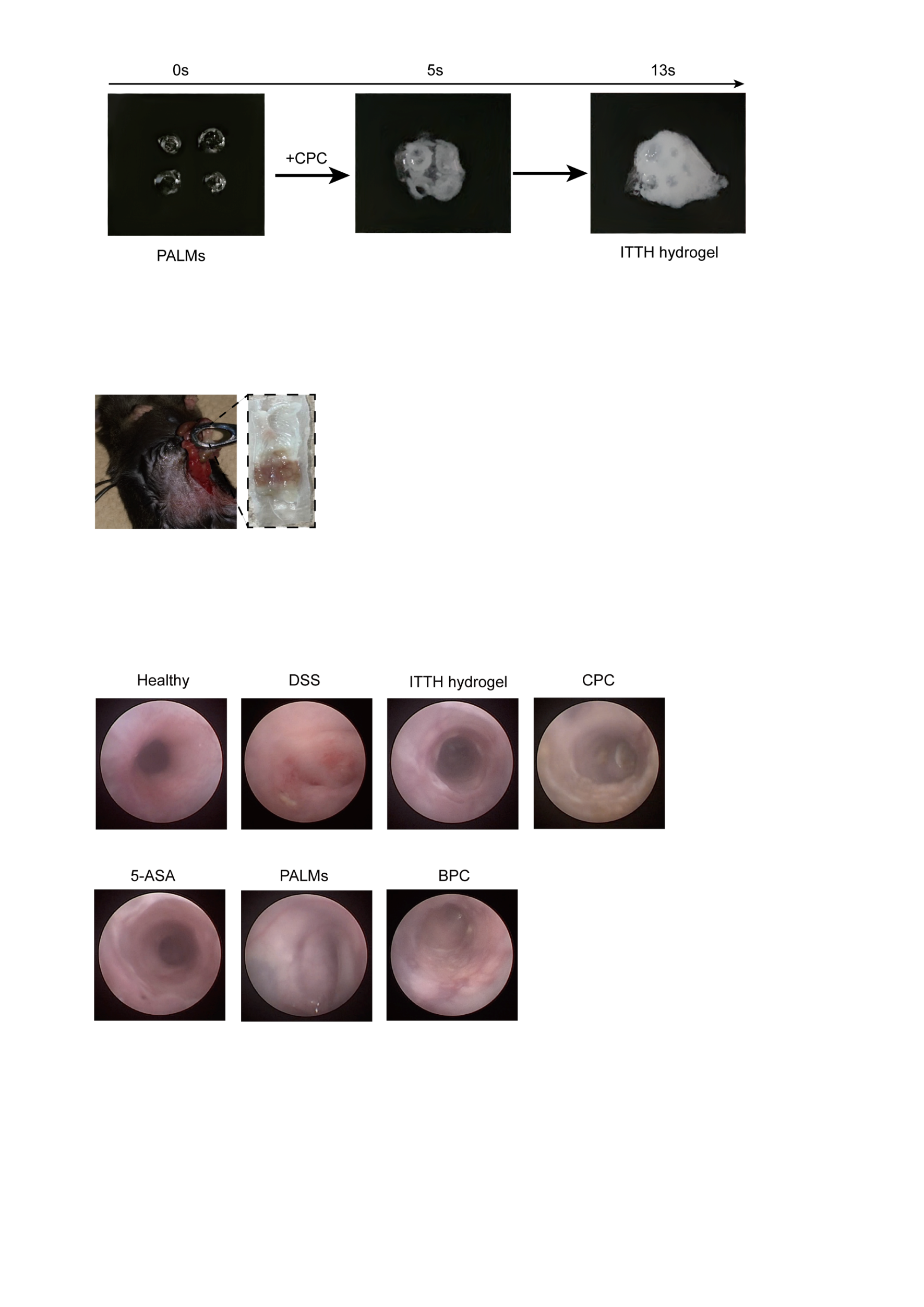
**

**Figure S8.** Fixed-point modeling of ITTH hydrogel application in live mice. Schematic diagram illustrating the procedure for fixed-point modeling in live mice, used to evaluate ITTH hydrogel adhesion and therapeutic efficacy at inflamed sites.


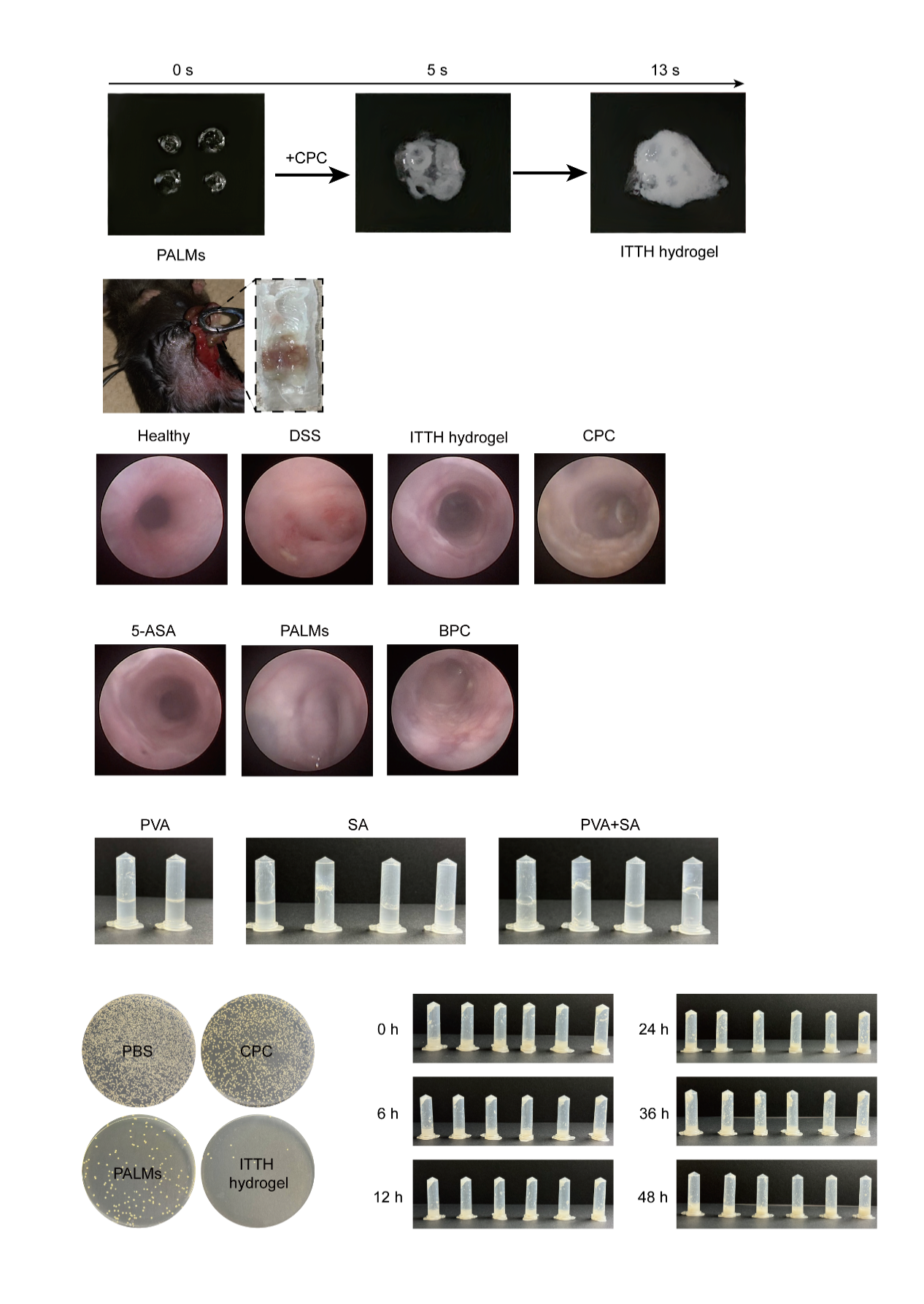


**Figure S9.** Permeability assessment of ITTH hydrogel against *S. aureus*. Photographic results of *S. aureus* permeability experiments showing bacterial penetration in samples treated with PBS, CPC, PALMs, and ITTH hydrogel. Reduced colony formation in the ITTH hydrogel sample demonstrates its barrier effectiveness.

**
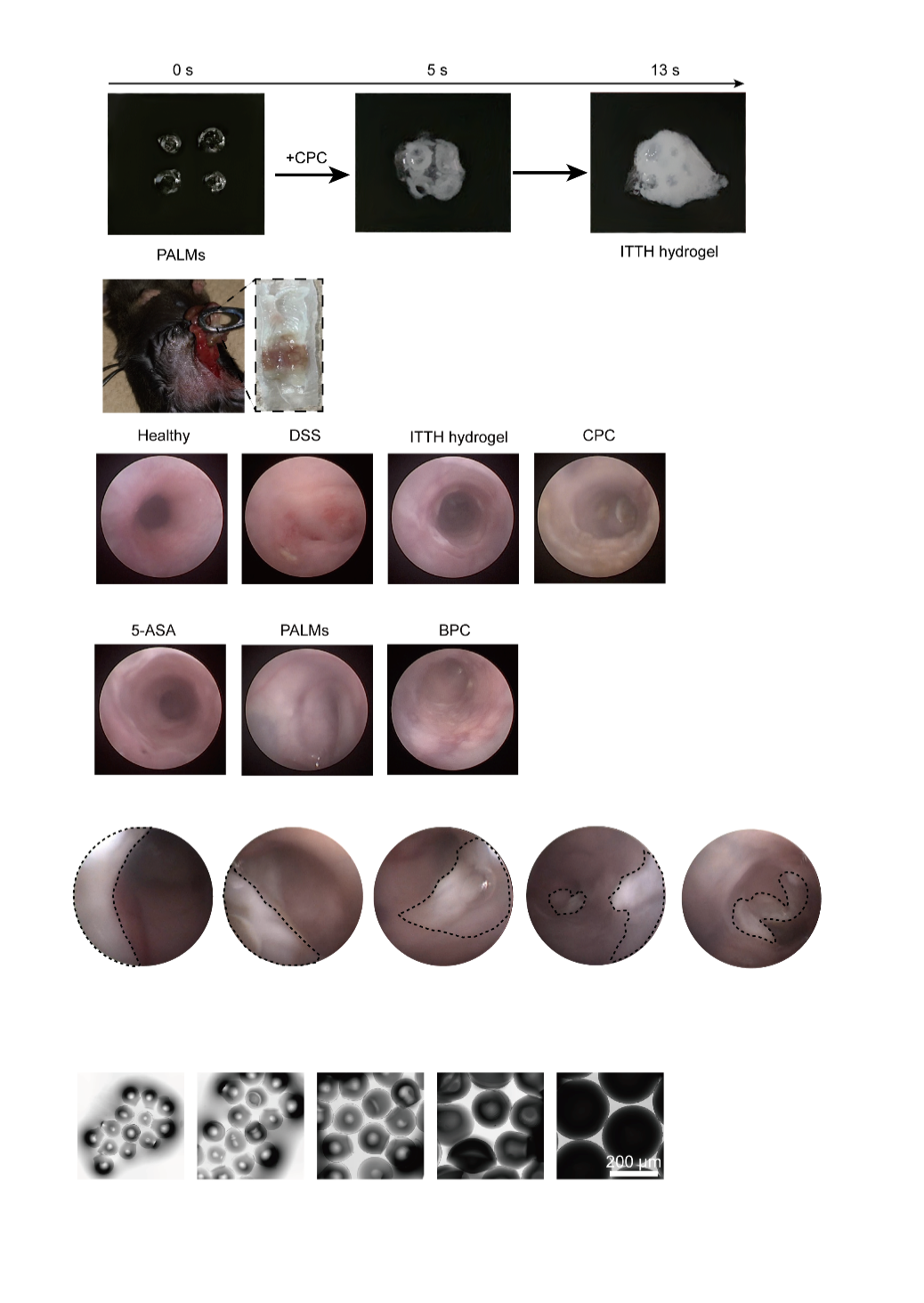
**

**Figure S10.** *In situ* cross-linking of ITTH hydrogel at an inflamed colonic site. Images showing ITTH hydrogel formation directly at an inflamed site in the colon, reconstructing the mucosal barrier. Black dotted areas highlight the extent of ITTH hydrogel coverage over the affected tissue.


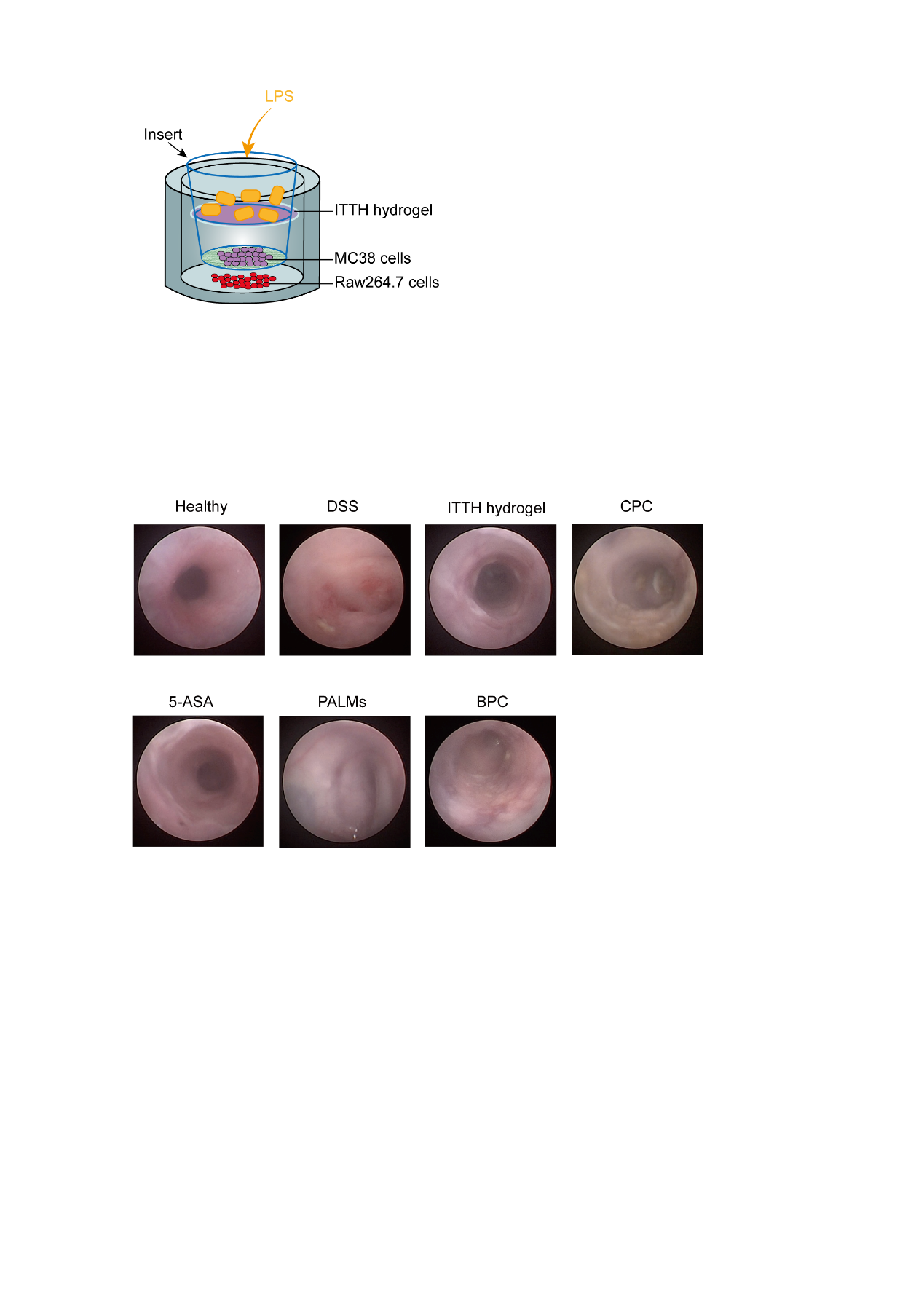


**Figure S11.** Endoscopic images of colon tissue post-treatment. Representative endoscopic images of the colon in various treatment groups, with dotted lines indicating the boundaries of healed or inflamed mucosal areas. These images illustrate the degree of mucosal recovery or remaining inflammation after ITTH hydrogel treatment.

**Movie S1.** Formation of ITTH hydrogel via secondary crosslinking. This video demonstrates the formation of the ITTH hydrogel. Upon adding the CPC to the PALMs microgel suspension, a second crosslinking occurs, resulting in the transformation of the suspension into a cohesive ITTH gelation network.

**Movie S2.** Colonoscopy shows specific adhesion of ITTH hydrogel without obstruction. One day after the administration of the ITTH hydrogel via enema, a colonoscopy was performed. The video shows the intestine with specific adhesion of the ITTH hydrogel to the inflamed mucosal sites, with no evidence of luminal obstruction.
